# Supplementary material for: Differences and agreement between two portable hand-held spirometers across diverse community-based populations in the Prospective Urban Rural Epidemiology (PURE) study
Source: PLOS Glob Public Health. 2022 Feb 2;2(2):e0000141. doi: 10.1371/journal.pgph.0000141 (PMC10021326; doi:10.1371/journal.pgph.0000141)
Supplement: S1 File — (DOC) [file pgph.0000141.s001.doc]

**APPENDIX I**

**Funding/Support:**

Dr S Yusuf is supported by the Marion W Burke endowed chair of the Heart and Stroke Foundation of Ontario.

The PURE study is an investigator-initiated study that is funded by the Population Health Research Institute, Hamilton Health Sciences Research Institute (HHSRI), the Canadian Institutes of Health Research, Heart and Stroke Foundation of Ontario, Support from Canadian Institutes of Health Research’s Strategy for Patient Oriented Research, through the Ontario SPOR Support Unit, as well as the Ontario Ministry of Health and Long-Term Care and through unrestricted grants from several pharmaceutical companies [with major contributions from AstraZeneca (Canada), Sanofi-Aventis (France and Canada), Boehringer Ingelheim (Germany and Canada), Servier, and GlaxoSmithKline], and additional contributions from Novartis and King Pharma and from various national or local organizations in participating countries.

These include: **Argentina:** Fundacion ECLA **(Estudios Clínicos Latino America)** ; **Bangladesh**: Independent University, Bangladesh and Mitra and Associates; **Brazil:** Hospital Alemão Oswaldo Cruz, São Paulo, Brazil; **Canada:** This study was supported by an unrestricted grant from Dairy Farmers of Canada and the National Dairy Council (U.S.), Public Health Agency of Canada and Champlain Cardiovascular Disease Prevention Network; **Chile:** Universidad de La Frontera [DI13-PE11]; **China:** National Center for Cardiovascular Diseases and ThinkTank Research Center for Health Development; **Colombia:** Colciencias (grant 6566-04-18062 and grant 6517-777-58228); **India:** Indian Council of Medical Research; **Malaysia:** Ministry of Science, Technology and Innovation of Malaysia (grant number: 100-IRDC/BIOTEK 16/6/21 [13/2007], and 07-05-IFN-BPH 010), Ministry of Higher Education of Malaysia (grant number: 600-RMI/LRGS/5/3 [2/2011]), Universiti Teknologi MARA, Universiti Kebangsaan Malaysia (UKM-Hejim-Komuniti-15-2010); **occupied Palestinian territory:** the United Nations Relief and Works Agency for Palestine Refugees in the Near East, occupied Palestinian territory; International Development Research Centre, Canada; **Philippines:** Philippine Council for Health Research and Development; **Poland:** Polish Ministry of Science and Higher Education (grant number: 290/W-PURE/2008/0), Wroclaw Medical University; **Saudi Arabia:** Saudi Heart Association, Dr.Mohammad Alfagih Hospital, The Deanship of Scientific Research at King Saud University (Research group number: RG -1436-013), Riyadh; Saleh Hamza Serafi Chair for Research of Coronary Heart Disease, Umm AlQura University, Makkah, Saudi Arabia; **South Africa:** The North-West University, SA and Netherlands Programme for Alternative Development, National Research Foundation, Medical Research Council of South Africa, The South Africa Sugar Association, Faculty of Community and Health Sciences; **Sweden:** Grants from the Swedish state under the Agreement concerning research and education of doctors; the Swedish Heart and Lung Foundation; the Swedish Research Council; the Swedish Council for Health, Working Life and Welfare, King Gustaf V:s and Queen Victoria Freemason’s Foundation, AFA Insurance; **Turkey:** Metabolic Syndrome Society, AstraZeneca, Sanofi Aventis; **United Arab Emirates:** Sheikh Hamdan Bin Rashid Al Maktoum Award For Medical Sciences and Dubai Health Authority, Dubai.

**Role of Sponsor:** The external funders and sponsors had no role in the design and conduct of the study; in the collection, analysis, and interpretation of the data; in the preparation, review, or approval of the

manuscript; or in the decision to submit the manuscript for publication.

**PURE Project Office Staff, National Coordinators, Investigators, and Key Staff:**

**Project office (Population Health Research Institute, Hamilton Health Sciences and McMaster University, Hamilton, Canada):** S Yusuf* (Principal Investigator).

S Rangarajan (Program Manager); K K Teo, S S Anand, C K Chow, M O’Donnell, A Mente, D Leong, A Smyth, P Joseph, M Duong, R D’Souza, M Walli-Attaei, S Islam (Statistician), W Hu (Statistician), C Ramasundarahettige (Statistician), P Sheridan (Statistician), S Bangdiwala, L Dyal, B Liu (Biometric Programmer), C Tang (Biometric Programmer), X Yang (Biometric Programmer), R Zhao (Biometric Programmer), L Farago (ICT), M Zarate (ICT), J Godreault (ICT), M Haskins (ICT), M Jethva (ICT), G Rigitano (ICT), A Vaghela (ICT), M Dehghan (Nutrition Epidemiologist), A Aliberti, A Reyes, A Zaki, B Connolly, B Zhang, D Agapay, D Krol, E McNeice, E Ramezani, F Shifaly, G McAlpine, I Kay, J Rimac, J Swallow, M Di Marino, M Jakymyshyn, M(a) Mushtaha, M(o) Mushtaha, M Trottier, N Aoucheva, N Kandy, P Mackie, R Buthool, R Patel, R Solano, S Gopal, S Ramacham, S Trottier

**Core Laboratories**: G Pare, M McQueen, S Lamers, J Keys (Hamilton), X Wang (Beijing, China), A Devanath (Bangalore, India).

**Argentina:** R Diaz*, A Orlandini, P Lamelas, M L Diaz, A Pascual, M Salvador, C Chacon; **Bangladesh:** O Rahman*, R Yusuf*, S A K S. Ahmed, T Choudhury, M Sintaha, A Khan, O Alam, N, Nayeem, S N Mitra, S Islam, F Pasha; **Brazil:** A Avezum*, C S Marcilio, A C Mattos, G B Oliveira; **Canada:**  K Teo***,** S Yusuf*****, Sumathy Rangarajan, A Arshad, B Bideri, I Kay, J Rimac, R Buthool, S Trottier, G Dagenais, P Poirier, G Turbide, AS Bourlaud, A LeBlanc De Bluts, M Cayer, I Tardif, M Pettigrew, S Lear, V de Jong, A N Saidy, V Kandola, E Corber, I Vukmirovich, D Gasevic, A Wielgosz, A Pipe, A Lefebvre, A Pepe, A Auclair, A Prémont, A S Bourlaud; **Chile:** F Lanas*, P Serón, M J Oliveros, F Cazor, Y Palacios; **China:** Liu Lisheng*, Li Wei*, Chen Chunming^#^, Zhao Wenhua. Hu Bo, Yin Lu, Zhu Jun, Liang Yan, Sun Yi, Wang Yang, Deng Qing, Jia Xuan, He Xinye, Zhang Hongye, Bo Jian, Wang Xingyu, Liu Xu, Gao Nan, Bai Xiulin, Yao Chenrui, Cheng Xiaoru, Wang Chuangshi, Li Sidong, Liu Weida, Lang Xinyue, Liu Xiaoyun, Zhu Yibing, Xie Liya, Liu Zhiguang, Ren Yingjuan, Dai Xi, Gao Liuning, Wang Liping, Su yuxuan, Han Guoliang, Song Rui, Cao Zhuangni, Sun Yaya, Li Xiangrong, Wang Jing, Wang Li, Peng Ya, Li Xiaoqing, Li Ling, Wang Jia, Zou Jianmei, Gao Fan, Tian Shaofang, Liu Lifu, Li Yongmei, Bi Yanhui, Li Xin, Zhang Anran, Wu Dandan, Cheng ying, Xiao Yize, Lu Fanghong, Li Yindong, Hou Yan, Zhang Liangqing, Guo Baoxia, Liao Xiaoyang, Chen Di, Zhang Peng, Li Ning, Ma Xiaolan, Lei Rensheng, Fu Minfan, Liu Yu, Xing Xiaojie, Yang Youzhu, Zhao Shenghu, Xiang Quanyong, Tang Jinhua, Liu Zhengrong, Qiang Deren, Li Xiaoxia, Xu Zhengting, Aideeraili.Ayoupu, Zhao Qian; **Colombia:** P Lopez-Jaramillo*, P A Camacho-Lopez, M Perez, J Otero-Wandurraga, D I Molina, C Cure-Cure, JL Accini, E Hernandez, E Arcos, C Narvaez, A Sotomayor, F Manzur, H Garcia, G Sanchez, F Cotes, A Rico, M Duran, C Torres; **India: Bangalore -** P Mony *, M Vaz*, S Swaminathan, AV Bharathi, K Shankar, A V Kurpad, K G Jayachitra, H A L Hospital, AR Raju, S Niramala, V Hemalatha, K Murali, C Balaji, A Janaki, K Amaranadh, P Vijayalakshmi, **Chennai** - V Mohan*, R M Anjana, M Deepa, K Parthiban, L Dhanasekaran, SK Sundaram, M Rajalakshmi, P Rajaneesh, K Munusamy, M Anitha, S Hemavathy, T Rahulashankiruthiyayan, D Anitha, R. Dhanasekar, S. Sureshkumar, D Anitha, K Sridevi, **Jaipur** - R Gupta, R B Panwar, I Mohan, P Rastogi, S Rastogi, R Bhargava, M Sharma, D Sharma, **Trivandrum** - V Raman Kutty, K Vijayakumar, S Nair, Kamala R, Manu MS, Arunlal AR, Veena A, Sandeep P Kumar, Leena Kumari, Tessi R, Jith S, K Ajayan, G Rajasree, AR Renjini, A Deepu, B Sandhya, S Asha, H S Soumya, **Chandigarh**- R Kumar, M Kaur, P V M Lakshmi, V Sagar J S Thakur, B Patro, R Mahajan, A Josh, G Singh, K Sharma, P Chaudary, **Iran:** R Kelishadi*, A Bahonar, N Mohammadifard, H Heidari, **Kazakhstan:** K Davletov*, B Assembekov, B Amirov; **Kyrgyzstan:** E Mirrakhimov*, S Abilova, U Zakirov, U Toktomamatov; **Malaysia: UiTM -** K Yusoff*, T S Ismail, K Ng, A Devi, N Mat-Nasir, AS Ramli, MNK Nor-Ashikin, R Dasiman, MY Mazapuspavina, F Ariffin, M Miskan, H Abdul-Hamid, S Abdul-Razak, N Baharudin, NMN Mohd-Nasir, SF Badlishah-Sham, MS Mohamed-Yassin, M Kaur, M Koshy, F A Majid, N A Bakar, N Zainon, R Salleh, SR Norlizan, NM Ghazali, M Baharom, H Zulkifli, R Razali, S Ali, CWJCW Hafar, F Basir; **UKM** - Noorhassim Ismail, M J Hasni, M T Azmi, M I Zaleha, R Ismail, K Y Hazdi, N Saian, A Jusoh, N Nasir, A Ayub, N Mohamed, A Jamaludin, Z Rahim; **Occupied Palestinian Territory:** R Khatib*, U Khammash, R Giacaman; **Pakistan:** R Iqbal*, R Khawaja, I Azam, K Kazmi; **Peru:** J Miranda*, A Bernabe Ortiz, W Checkley, R H Gilman, L Smeeth, R M Carrillo, M de los Angeles, C Tarazona Meza**;** **Philippines:** A Dans*, H U Co, J T Sanchez, L Pudol, C Zamora-Pudol, L A M Palileo-Villanueva, M R Aquino, C Abaquin, SL Pudol, K Manguiat, S Malayang; **Poland:** W Zatonski*, A Szuba, K Zatonska, R Ilow**^#^**, M Ferus, B Regulska-Ilow, D Różańska, M Wolyniec; **Saudi Arabia:** KF AlHabib*, M Alshamiri, HB Altaradi, O Alnobani, N Alkamel, M Ali, M Abdulrahman, R Nouri; **South Africa:** L Kruger^*^, A Kruger^#^, P Bestra, H Voster, A E Schutte, E Wentzel-Viljoen, FC Eloff, H de Ridder, H Moss, J Potgieter, A Roux, M Watson, G de Wet, A Olckers, J C Jerling, M Pieters, T Hoekstra, T Puoane, R Swart*, E Igumbor, L Tsolekile, K Ndayi, D Sanders, P Naidoo, N Steyn, N Peer, B Mayosi^#^, B Rayner, V Lambert, N Levitt, T Kolbe-Alexander, L Ntyintyane, G Hughes, J Fourie, M Muzigaba, S Xapa, N Gobile , K Ndayi, B Jwili, K Ndibaza, B Egbujie; **Sweden** A Rosengren*, K Bengtsson Boström, A Rawshani, A Gustavsson, M Andreasson, L Wirdemann; **Tanzania:** K Yeates*, M Oresto, N West **Turkey:** A Oguz*, N Imeryuz, Y Altuntas, S Gulec, A Temizhan, K Karsidag, K B T Calik, A K Akalin, O T Caklili, M V Keskinler, K Yildiz; **United Arab Emirates:** A H Yusufali, F Hussain, M H S Abdelmotagali, D F Youssef, O Z S Ahmad, F H M Hashem, T M Mamdouh, F M AbdRabbou, S H Ahmed, M A AlOmairi, H M Swidan, M Omran, N A Monsef ; **Zimbabwe:** J Chifamba*, T Ncube, B Ncube, C Chimhete, G K Neya, T Manenji, L Gwaunza, V Mapara, G Terera, C Mahachi, P Murambiwa, R Mapanga, A Chinhara

*National Coordinator

^#^ Deceased

**PURE Country Institution Names:**

|  | **Institution** |
| --- | --- |
| **South Africa** | Faculty of Health Science, North-West University  Potchefstroom Campus |
|  | University of the Western Cape, Department of Dietetics and Nutrition  Private Bag X17, 7535  Bellville, South Africa |
| **Zimbabwe** | University of Zimbabwe, College of Health Sciences, Physiology Department  Harare, Zimbabwe |
| **Tanzania** | Pamoja Tunaweza Health Research Centre, Moshi, Tanzania  Division of Nephrology, Department of Medicine  Queen's University |
| **China** | National Centre for Cardiovascular Diseases, Cardiovascular Institute & Fuwai Hospital, Chinese Academy of Medical Sciences  167, Bei Li Shi Lu, Beijing, China |
|  | Fuwai Hospital  167 Beilishi Rd. Xicheng District  Beijing. 100037 China |
| **Philippines** | University of Philippines, Section of Adult Medicine & Medical Research Unit, Manila, Philippines |
| **Pakistan** | Department of Community Health Sciences and Medicine, Aga Khan University  Stadium Road, P.O Box 3500  Karachi Pakistan |
| **India, Bangalore** | St John's Medical College and Research Institute Bangalore 560034, India |
| **India, Chennai** | Madras Diabetes Research Foundation &  Dr. Mohan’s Diabetes Specialities Centre, Chennai |
| **India Jaipur** | Eternal Heart Care Centre and Research Institute, Jaipur |
| **India, Trivandrum** | Health Action by People,  Thiruvananthapuram, Kerala, 695011 INDIA |
| **India, Chandigarh** | School of Public Health, Post Graduate Institute of Medical Education & Research, Chandigarh (India) |
| **Bangladesh** | Independent University, Bangladesh  Bashundhara, Dhaka, Bangladesh |
| **Malaysia** | Universiti Teknologi MARA, Sungai Buloh, Selangor, Malaysia AND UCSI University, Cheras, Selangor, Malaysia |
|  | Department of Community Health. Faculty of Medicine. University Kebangsaan Malaysia. Kuala Lumpur. Malaysia |
| **Poland** | Wroclaw Medical University Department of Internal Medicine; Department of Social Medicine Borowska 213 street; 50- 556 Wroclaw, Poland |
|  | Department of Epidemiology,  The Maria Skłodowska-Curie Memorial Cancer Center and Institute of Oncology, 02-034 Warsaw, 15B Wawelska str.  Poland |
| **Turkey** | Istanbul Medeniyet University  Istanbul, Turkey |
| **Sweden** | Sahlgrenska Academy  University of Gothenburg  Sweden |
| **Iran** | Isfahan Cardiovascular Research Center, Isfahan Research Institute  Isfahan University of Medical Sciences, Isfahan, Iran |
| **UAE** | Dubai Medical University, Hatta Hospital, Dubai Health Authority, Dubai, United Arab Emirates |
| **Saudi Arabia** | Department of Cardiac Sciences, King Fahad Cardiac Center  College of Medicine, King Saud University  Riyadh, Saudi Arabia |
| **Palestine** | Institute of Community and Public Health, Birzeit University, Ramallah, occupied Palestinian territory |
| **Canada** | Université Laval Institut universitaire de cardiologie et de pneumologie de Québec, Quebec  Canada G1V 4G5 |
|  | Simon Fraser University,  Dept. of Biomedical Physiology & Kinesiology, BC, Canada |
|  | Department of Medicine, University of Ottawa,  Ottawa, Canada |
|  | Population Health Research Institute, McMaster University, Hamilton Health Sciences, Hamilton, Ontario, Canada |
| **Argentina** | Estudios Clinicos Latinoamerica ECLA  Rosario, Santa Fe  Argentina  Department of Chronic Diseases  South American Center of Excellence for Cardiovascular Health (CESCAS)  Institute for Clinical Effectiveness and Health Policy (IECS) |
| **Brazil** | Dante Pazzanese Institute of Cardiology;  Hospital Alemao Oswaldo Cruz  Sao Paulo, SP Brazil |
| **Colombia** | Facultad de Ciencias de la Salud, Universidad de Santander (UDES), Bucaramanga, Santander, Fundacion Oftalmologica de Santander (FOSCAL)  Floridablanca-Santander, Colombia |
| **Chile** | Universidad de La Frontera  Temuco, Chile |
| **Ecuador** | DECANO  Facultad de Ciencias de la Salud Eugenio Espejo  Universidad Tecnológica Equinoccial  Dirección: Av. Mariscal Sucre s/n y Av. Mariana de Jesús, Quito Ecuador |
| **Peru** | CRONICAS Centro de Excelencia en Enfermedades Crónicas | [www.cronicas-upch.pe](http://www.cronicas-upch.pe/)  Universidad Peruana Cayetano Heredia | www.upch.edu.pe  Av. Armendáriz 497, Miraflores, Lima |
| **Russia** | Research Institute for Complex Issues of Cardiovascular Diseases, Kemerovo, Russia  Institute For Medical Education, Yaroslav-the-Wise Novgorod State University Ministry of Education and Science of the Russian Federation  Russia, Saint-Petersburg, 197022,  Karpovka river emb., Bld.13, office 28 |
| **Kazakhstan** | Research Institute of Cardiology & Internal Diseases, Almaty, Kazakhstan |
| **Kyrgyzstan** | Kyrgyz Society of Cardiology, National Center of Cardiology and Internal Disease, Bishkek, Kyrgyzstan |

**Names of each ethics review board and institution that approved the PURE study.**

| Country | Name of ethics review board and institution |
| --- | --- |
| Argentina | Comité de Etica en Investigación Clínica (CEIC) |
| Brazil | Comissão Nacional de Ética em Pesquisa – Conep approval nº 9047; Comitê de Ética do Instituto Dante Pazzanese de Cardiologia – CEP/Dante approva nº 3310  Institution – HOSPITAL ALEMAO OSWALDO CRUZ |
| Chile | Comite de Etica Científica del Servicio de Salud Araucanía Sur |
| Colombia | Comité de Ética en Investigación de la Fundación Cardiovascular de Colombia |
| Canada – Hamilton | Hamilton Integrated Research Ethics Board  REB Project # 03-206 |
| Canada – Quebec | Comité d’éthique de la recherche de l’Institut Universitaire de Cardiologie et de Pneumologie de Québec |
| Canada – Ottawa | Ottawa Hospital Research Ethics Boards |
| Canada – Vancouver | Office of Research Ethics, Simon Fraser University |
| Sweden | Regional Ethical Review Board of Gothenburg |
| Poland | Institutional review boards at Medical University of Wroclaw |
| Turkey | Marmara University Medical Faculty Ethics Committee-2005  HA Istanbul Göztepe Training and Research Hospital Ethics Committee -2007  Health Authority approval-2008 |
| Iran | Ethics Committee, Isfahan Cardiovascular Research Center, Isfahan University of Medical Sciences |
| UAE | Medical Research Committee, Dubai Health Authority. |
| S Africa – Potchefstroom | Faculty of Health Sciences: Ethics sub-committee (Sub-committee of the North-West University Research Ethics Committee) |
| S Africa – Cape Town | Senate Research Committee of the University of the Western Cape, South Africa |
| Zimbabwe | Joint Ethics Research Committee (JREC)  Medical Research Council of Zimbabwe (MRCZ) |
| China | Beijing Hypertension League Institute Ethics Committee |
| Malaysia – UiTM | Research Ethics Committee UiTM, Malaysia |
| Malaysia – UKM | Research Ethics Committee., Universti Kebangsaan Malaysia, Medical Centre, Kuala Lumpur, Malaysia |
| India – Bangalore | St John's Medical College & Hospital Institutional Ethical Review Board |
| India – Jaipur | Institutional Ethics Committee of Monilek Hospital and Research Centre, Jaipur |
| India – Chennai | Institutional Ethics Committee of Madras Diabetes Research Foundation |
| India – Trivandrum | Ethics Committee, Health Action by People |
| India – Chandigarh | Institute Ethics Committee, Post Graduate Institute of Medical Education and Research (PGIMER), Chandigarh |
| Pakistan | Ethical Review Committee. The Aga Khan University |
| Bangladesh | Bangladesh Medical Research Council |
| Tanzania | The National Institute for Medical Research Tanzania (NIMR) |
| Saudi Arabia | King Saud University Medical City IRB |

**APPENDIX II: Baseline characteristics of the larger PURE study excluding participants from the current substudy**.

|  | **Overall** | **S Asia** | **China** | **S East Asia** | **Africa** | **Middle East** | **S America** | **N Am/Eur** |
| --- | --- | --- | --- | --- | --- | --- | --- | --- |
| **N** (total) | 94,917 (100) | 15,171 (16) | 24,457 (25.8) | 8,732 (9.2) | 3,638 (3.8) | 11,999 (12.6) | 16,124 (17) | 14,796 (15.6) |
| **Females** | 56,181 (59.2) | 8,870 (58.5) | 14,269 (58.3) | 5,557 (63.6) | 2,708 (74.5) | 6,602 (55.0) | 10,091 (62.6) | 8,084 (54.6) |
| **Urban** | 49,052 (51.7) | 6,130 (40.4) | 10,848 (44.4) | 3,112 (35.6) | 1,495 (41.1) | 6,696 (55.8) | 9,741 (60.4) | 11,030 (74.6) |
| **Age,** years | 50.5 ± 9.7 | 47.1 ± 9.6 | 51.3 ± 9.5 | 52.4 ± 9.3 | 50.4 ± 10.1 | 48.7 ± 9.1 | 51.3 ± 9.5 | 53.4 ± 9.1 |
| **Weight**, (kg) | 68.5 ± 16.6 | 57.8 ± 13.4 | 63.7 ± 12.3 | 62.7 ± 14.0 | 68.2 ± 18.9 | 75.7 ± 15.2 | 74.1 ± 16.5 | 78.2 ± 17.1 |
| **BMI** (kg/m^2^) | 26.3 ± 5.4 | 23.1 ± 4.7 | 24.5 ± 3.7 | 25.6 ± 4.9 | 26.5 ± 7.0 | 28.9 ± 5.5 | 28.4 ± 5.5 | 27.3 ± 5.1 |
| **Height** (cm) | 161.4 ± 9.7 | 157.9 ± 8.9 | 160.6 ± 8.3 | 156.1 ± 8.2 | 160.2 ± 8.0 | 161.9 ± 9.5 | 161.2 ± 9.5 | 168.8 ± 9.6 |
| **COPD/asthma** | 4,190 (4.4) | 456 (3.0) | 553 (2.3) | 385 (4.4) | 145 (4.1) | 690 (5.8) | 705 (4.4) | 1,256 (8.5) |
| **Cardiac disease** | 3,432 (3.6) | 238 (1.6) | 1,272 (5.2) | 152 (1.8) | 145 (4.0) | 580 (4.8) | 448 (2.8) | 597 (4.0) |
| **Strokes** | 1,447 (1.5) | 93 (0.6) | 572 (2.4) | 169 (1.9) | 57 (1.6) | 143 (1.2) | 224 (1.4) | 191 (0.2) |
| **Smoked tobacco** current  ex-smokers  never | 18,580 (19.7)  12,329 (13.1)  63,195 (67.2) | 3,247 (21.5)  603 (4.0)  11,226 (74.5) | 5,325 (22.2)  1,017 (4.2)  17,679 (73.6) | 1,143 (13.2)  795 (9.2)  6,727 (77.6) | 749 (21.2)  171 (4.8)  2,610 (73.9) | 2,321 (19.3)  1,042 (8.7)  8,636 (71.9) | 3,563 (22.2)  3,512 (21.9)  8,973 (55.9) | 2,232 (15.1)  5,189 (35.1)  7,344 (49.7) |
| **Education**  Primary/below  Secondary/above | 39,691 (41.9)  54,782 (57.9) | 7,345 (48.7)  7,697 (51.0) | 9,059 (37.1)  15,312 (62.8) | 3,433 (39.3)  5,260 (60.3) | 2,366 (65.5)  1,195 (33.1) | 7,126 (59.4)  4,865 (40.6) | 9,309 (57.9)  6,753 (42.0) | 1,053 (7.1)  13,700 (92.7) |
| **Grades A-B*** | 29,191 (48.7) | 3,741 (36.5) | 4,210 (51.3) | 2,743 (43.5) | 934 (32.5) | 4,442 (41.6) | 4,143 (43.4) | 8,978 (74.3) |
| **Grade C*** | 9,917 (16.5) | 1,771 (17.3) | 1,575 (19.2) | 1,229 (19.5) | 480 (16.7) | 1,857 (17.4) | 1,552 (16.3) | 1,453 (12) |
| **Grade D*** | 14,620 (24.4) | 2,975 (29) | 2,047 (24.9) | 1,534 (24.4) | 1,020 (35.4) | 3,168 (29.7) | 2,480 (26) | 1,396 (11.6) |
| **Grade F*** | 6095 (10.2) | 1,743 (17) | 330 (4) | 792 (12.6) | 418 (14.5) | 1,202 (11.3) | 1,368 (14.3) | 242 (2) |

Data are presented as means ± SD for continuous data and absolute numbers (% of total in each region/ column). Abbreviations: BMI=body mass index calculated as weight divided by height squared; COPD (chronic obstructive pulmonary disease)/asthma, cardiac disease and strokes were based on self-reported response to baseline interview-based questionnaire; *the quality grades using ATS/ERS guidelines were based on 59,823 participants with follow-up data from the EasyOne spirometer. For regions S=South; N Am/Eur=North America/Europe.

**APPENDIX III: Baseline characteristics of participants within and outside the LoA for the overall study.**

|  | **Overall** | **FEV_1_: within LoA** | **FEV_1_: outside LoA** | **FVC: within LoA** | **FVC: outside LoA** |
| --- | --- | --- | --- | --- | --- |
| N (total) | 4603 | 4293 | 310 | 4275 | 299 |
| Females | 2840 (61.7) | 2673 (62.3) | 167 (53.9) | 2637 (61.6) | 203 (63.4) |
| Urban | 2363 (51.4) | 2204 (51.4) | 159 (51.3) | 2196 (51.3) | 167 (52.2) |
| Age, years | 58.5 ±10.2 | 58.4 ±9.9 | 58.9 ±13.3 | 58.5 ±10.2 | 58.2 ±10.3 |
| Weight, kg | 70.2 ±16.0 | 70.3 ±16.0 | 68.8 ±15.9 | 70.4 ±16.0 | 67.7 ±16.1 |
| BMI, kg/m^2^ | 27.2 ±5.5 | 27.2 ±5.5 | 26.7 ±5.5 | 27.2 ±5.5 | 26.9 ±5.8 |
| Height, cm | 160.6 ±9.8 | 160.6 ±9.7 | 160.4 ±11.2 | 160.7 ±9.8 | 158.6 ±9.9 |
| COPD/asthma | 293 (6.4) | 282 **(6.6)** | 10 (3.2) | 277 (6.5) | 16 (5.0) |
| cardiac disease | 253 (5.5) | 232 **(5.4)** | 21 (6.8) | 232 (5.4) | 21 (6.6) |
| Strokes | 110 (2.4) | 105 **(2.5)** | 5 (1.6) | 102 (2.4) | 8 (2.5) |
| Smoked tobacco Current  Ex-smokers  Never | 954 (20.8)  628 (13.7)  3013 (65.6) | 905 **(21.1)**  579 (13.5)  2801 (65.4) | 49 (15.8)  49 (15.8)  212 (68.4) | 901 **(21.1)**  585 (13.7)  2789 (65.2) | 53 (16.6)  43 (13.4)  224 (70) |
| Education  Primary/ below  Secondary/ above | 2368 (51.7)  2213 (48.3) | 2178 (51.0)  2094 (49.0) | 190 **(61.5)**  119 **(38.5)** | 2170 (50.9)  2092 (49.1) | 198 (62.1)  121 (37.9) |
| Grades A-B | 2172 (47.3) | 2087 (48.6) | 85 (27.6) | 2083 (48.7) | 89 (28.0) |
| Grade C | 783 (17.0) | 740 (13.7) | 43 (14.0) | 739 (17.3) | 44 (13.8) |
| Grade D/F | 1641 (35.7) | 1461 (34.1) | 180 **(58.4)** | 1456 (34.0) | 185 **(58.2)** |

Data are presented as mean ± SD for continuous variables and frequency (% of total N in column). COPD (chronic obstructive pulmonary disease)/asthma, cardiac disease and strokes were self-reported conditions to baseline questionnaire. Grades are quality grade for spirometry effort provided by the EasyOne spirometer.

**APPENDIX IV: Stratified analyses by demographic, anthropometric, clinical characteristics and quality grades.** These graphs depict the findings presented in Table 3 of the main paper.

**Fig A:** Scatterplots of correlation (95% CI) and mean differences (5-95 percentile) between paired FEV_1_ and FVC from different spirometers stratified by sex.

**Fig B:** Scatterplots of correlation (95% CI) and mean differences (5-95 percentile) between paired FEV_1_ and FVC from different spirometers stratified by age.

**Fig C:** Scatterplots of correlation (95%CI) and mean differences (5-95 percentile) between paired FEV_1_ and FVC from different spirometers stratified by BMI.

**Fig D:** Scatterplots of correlation (95%CI) and mean differences (5-95 percentile) for paired FEV_1_ and FVC from different spirometers stratified by smoking status.

**Fig E:** Scatterplots of correlation (95%CI) and mean differences (5-95 percentile) between paired FEV_1_ and FVC from different spirometers stratified by self-reported COPD or asthma.

**Fig F:** Scatterplots of correlation (95% CI) and mean differences (and 5-95 percentile) between paired FEV_1_ and FVC from different spirometers stratified by education level.

**Fig G:** Scatterplots of correlation (95% CI) and mean differences (5-95 percentile) between paired FEV_1_ and FVC from different spirometers stratified by quality grades.
