# Supplementary material for: Differences and agreement between two portable hand-held spirometers across diverse community-based populations in the Prospective Urban Rural Epidemiology (PURE) study
Source: PLOS Glob Public Health. 2022 Feb 2;2(2):e0000141. doi: 10.1371/journal.pgph.0000141 (PMC10021326; doi:10.1371/journal.pgph.0000141)
Supplement: Attachment — Submitted filename: PLOSPH_ReviewerResponseNov2021.1.docx [file pgph.0000141.s002.docx]

**Reviewer #1**: Thank you very much for the opportunity to review this manuscript. Duong and colleagues assess the comparison between two different spirometers in a very diverse population and in a larger study. This contributes good evidence on comparisons of spirometers especially in a real world setting as previous comparisons have been small and in a controlled setting. 
Taking advantage of this large and diverse population, this reviewer feels the analyses can be expanded by comparing the two spirometers using the Global Lung Initiative reference equations. This would also help to provide very vital insight on how the GLI behave in this population as they have been indicated to behave differently.

- We thank the reviewer for the above suggestion. There are limited data to support the fit of GLI for some of the regions/ countries represented in PURE. These include South Asia, South East Asia, the Middle East and certain parts of Africa. Nonetheless, we have expanded the analysis to compare the 2 spirometers using the GLI references equations. Our findings showed that differences in Z scores across regions were <0.5 SD and therefore will not have any meaningful impact in the interpretation using GLI normative values. This finding has been added to the revised manuscript as Table 5 and also provided below.

**Table 5. Mean Differences in Z scores (using GLI normative values) and the 95% Limits of agreement (LoA) between spirometers.**

|  | **OVERALL** | **S Asia** | **China** | **SE Asia** | **Africa** | **Middle East** | **S America** | **NAm/Eur** |
| --- | --- | --- | --- | --- | --- | --- | --- | --- |
| **N** | 4574 | 550 | 630 | 191 | 368 | 428 | 1571 | 836 |
| **FEV_1_***  (95% LOA) | **-0.13**  (2.7, -3.0) | **-0.088**  (-3.6, 3.4) | **0.15**  (-1.9, 2.2) | **-0.021**  (-2.3, 2.3) | **-0.13**  (-2.9, 2.6) | **-0.22**  (-3.1, 2.7) | **-0.25**  (-3.6, 3.1) | **-0.10**  (-1.7, 1.4) |
| **FVC***  (95% LOA) | **0.073**  (-3.3, 3.4) | **0.055**  (-4.6, 4.7) | **0.17**  (-2.6, 2.9) | **-0.11**  (-2.5, 2.2) | **0.12**  (-3.0, 3.3) | **-0.49**  (-4.2, 3.2) | **0.31**  (-3.4, 4.0) | **-0.11**  (-1.8, 1.6) |

This reviewer is also interested in the risk exposure-Lung function associations. Were these associations adjusted or crude? Were the lung function measurements mutually adjusted for? Since one measurement was at baseline and another at follow up? More specific details on this part of the analysis would benefit the reader. It would also be interesting to know whether these risk exposure –lung function associations have been assessed independently at baseline and at follow up in the said population. It is less surprising that the similar effects were observed for both spirometers since relatively high correlations were observed and indications point that the use of a particular spirometer was not associated with a particular region/subset of the cohort. This would complicate the risk exposure-lung function relationships especially if particular regions have higher PM2.5 levels etc.

The risk exposure-Lung function analysis has been removed from this revised paper. This was an interim analysis from the larger PURE study and therefore the sample sizes for data collected using the old and new spirometer were not balanced. Furthermore the methodology for this analysis is not well detailed in the current paper and to add this information to the current paper will substantially increase the length of the paper. Finally, we are planning to submit the risk exposure-lung function association as a separate and full paper and therefore we want to avoid the risk of duplicate publication. For these reasons, we have removed this data from the revised version of this paper. However, we will leave it up to the discretion of the editor, to let us know if by adding this data will strengthen the current work, which has already been expanded to include other analyses in the revised form. If this is the case, we will be happy to add this data back into the paper.

**Reviewer #2:** Thank you for the opportunity to review this valuable sub study from an international multi-centre prospective cohort examining the reliability in measurements between two handheld spirometers. The study uses a large and diverse population to support reliability and generalisability, and presents further findings according to strata and pollution exposure. These findings will support the global health community in ensuring accessibility to lung function testing. I have a number of comments and suggestions for sensitivity and sub analysis that would support the authors’ message and reliability of evidence.

The authors report little effect between strata according to sex, age category, BMI category, smoking status, and COPD/Asthma however there are no tests reported to confirm lack of significant effect. Either statistical tests or visualisation of data would support the assertions of minimal variation across strata.

- The correlation between spirometers for paired FEV1 and FVC across strata by sex, age, BMI, smoking status, COPD/asthma, education level and quality grades have been plotted and provided in Appendix IV to help with the visualization of the data. Overall, there was good correlation and significant overlap in the mean differences between spirometers and in the distribution of the data across strata. The 2 exceptions were the trend to larger variability observed with low education level and poor quality grade efforts. These data have been added to Table 4 and Appendix IV.

Certain regions have lower percentages of high grade blows and high percentages of low grade blows. Do the authors have any insight into the rationale for differences in grade between regions? The authors could present supplemental sub analysis to investigate the potential source of variability, such as countries within region. There may be more consistent bias within specific countries, which may represent particular factors e.g. socioeconomics.

- As suggested by the reviewer, we conducted a random-intercept multilevel ‘null’ model to parse out the contribution to variability between spirometers at the region, country, center and participant/ individual levels. The findings are shown below and have been added to the revised manuscript as Table 3.

**Table 3. ICC and variance components between paired FEV_1_ and FVC at different levels according to region, country, centers and individuals.**

|  | **FEV_1_** | | **FVC** | |
| --- | --- | --- | --- | --- |
| **Levels** | **ICC** (95% CI) | **Variance** (95%CI), Litres^2^ | **ICC** (95% CI) | **Variance** (95%CI), Litres^2^ |
| Region | 0.133 (0.039, 0.361) | 0.084 (0.023, 0.306) | 0.146 (0.042, 0.399) | 0.148 (0.039, 0.559) |
| Country within region | 0.168 (0.069, 0.356) | 0.023 (0.003, 0.155) | 0.202 (0.087, 0.402) | 0.056 (0.011, 0.291) |
| Centers within countries | 0.208 (0.104, 0.373) | 0.025 (0.009, 0.012) | 0.248 (0.129, 0.422) | 0.046 (0.022, 0.096) |
| Individuals within centers | **0.783 (0.743, 0.819**) | **0.366 (0.348, 0.384)** | **0.730 (0.673, 0.781)** | **0.488 (0.463, 0.515)** |
| Residual error |  | 0.138 (0.132, 0.143) |  | 0.273 (0.262, 0.284) |

- As shown, the ICC or correlation between FEV_1_ across spirometers was highest at the participant level at 0.783, indicating that FEV_1_ between spirometers were highly correlated within individuals. Consistent with this, is the variance estimate, which was highest at the individual level, suggesting that inter-subject factors contribute significantly to the variability between spirometers. The correlations and contribution to variability between spirometers for the other levels were substantially less.
- The contribution to variability between spirometers by factors related to region was much smaller but meaningful (suggesting some variation among regions), while the variance components for country and center were smaller still, suggesting little variations between countries and between centers. Furthermore, the increase in correlation and variance estimates from region to country and to centers were not dramatic, compared to the large increase from center to individual level, highlighting the importance of individual factors contributing to the variability observed between spirometers. Similar findings were observed in the analyses for FVC comparing the two spirometers.

Can the authors report the % of blows that are outside the limits of agreement in the Bland Altman plots/table, and can the authors report any sub analyses that explore the characteristics of participants that were outside these limits compared to within to assess whether there are factors that bias reliability between spirometers. Similarly, it would be interesting to present the grade of EasyOne measures using colours on the Bland Altmann plots to interpret how grade may have contributed to agreement between spirometers.

- The number of individuals and the % of data, which fall outside the limits of agreement (LoA) are provided in the table below. We have also provided the baseline characteristics for participants, whose inter-device differences were within and outside of the overall population LoA in Appendix III. As shown, there were no differences in age, BMI and gender distribution between participants within and outside the LoA. Furthermore, having COPD/asthma, cardiac disease, strokes and tobacco smoking did not adversely impact the agreement between spirometers. However, there was a trend for lower quality grade efforts and lower education level in those outside the LoA. Next, we examined the mean differences and LoA using the Bland-Altman approach across the quality grade measurements (added to Table 4 of the revised manuscript). Our findings suggest, that while the LoA did increase with lower grades, mean differences between devices even for the lower grades D/F remained within the acceptable limits for within individual variability.

**Table A.** Number of participants (% of column) outside the LoA specific for overall population and by region.

|  | **OVERALL** | **S Asia** | **China** | **SE Asia** | **Africa** | **M East** | **S America** | **N Am / Eur** |
| --- | --- | --- | --- | --- | --- | --- | --- | --- |
| **N** | 4603 | 566 | 631 | 191 | 368 | 433 | 1578 | 836 |
| **FEV_1_, ml** | 299 (6.5%) | 45 (7.9%) | 38 (6.0%) | 7 (3.7%) | 17 (4.6%) | 26 (6.0%) | 112 (7.0%) | 34 (4.1%) |
| **FVC_,_ ml** | 304 (6.6%) | 48 (8.5%) | 40 (6.3%) | 3 (1.6%) | 23 (6.2%) | 27 (6.2%) | 110 (6.9%) | 37 (4.4%) |
| **FEV, %** | 333 (7.2%) | 38 (6.7%) | 38 (6.0%) | 7 (3.7%) | 30 (8.1%) | 27 (6.2%) | 116 (7.3%) | 32 (3.8%) |
| **FVC, %** | 323 (7.0%) | 45 (7.9%) | 34 (5.4%) | 4 (2.1%) | 25 (6.8%) | 24 (5.5%) | 124 (7.8%) | 40 (4.8%) |

**Table B.** Baseline characteristics of participants within and outside the LoA for the overall study.

|  | **Overall** | **FEV_1_: within LoA** | **FEV_1_: outside LoA** | **FVC: within LoA** | **FVC: outside LoA** |
| --- | --- | --- | --- | --- | --- |
| N (total) | 4603 | 4293 | 310 | 4275 | 299 |
| Females | 2840 (61.7) | 2673 (62.3) | 167 (53.9) | 2637 (61.6) | 203 (63.4) |
| Urban | 2363 (51.4) | 2204 (51.4) | 159 (51.3) | 2196 (51.3) | 167 (52.2) |
| Age, years | 58.5 ±10.2 | 58.4 ±9.9 | 58.9 ±13.3 | 58.5 ±10.2 | 58.2 ±10.3 |
| Weight, kg | 70.2 ±16.0 | 70.3 ±16.0 | 68.8 ±15.9 | 70.4 ±16.0 | 67.7 ±16.1 |
| BMI, kg/m^2^ | 27.2 ±5.5 | 27.2 ±5.5 | 26.7 ±5.5 | 27.2 ±5.5 | 26.9 ±5.8 |
| Height, cm | 160.6 ±9.8 | 160.6 ±9.7 | 160.4 ±11.2 | 160.7 ±9.8 | 158.6 ±9.9 |
| COPD/asthma | 293 (6.4) | 282 **(6.6)** | 10 (3.2) | 277 (6.5) | 16 (5.0) |
| cardiac disease | 253 (5.5) | 232 **(5.4)** | 21 (6.8) | 232 (5.4) | 21 (6.6) |
| Strokes | 110 (2.4) | 105 **(2.5)** | 5 (1.6) | 102 (2.4) | 8 (2.5) |
| Smoked tobacco Current  Ex-smokers  Never | 954 (20.8)  628 (13.7)  3013 (65.6) | 905 **(21.1)**  579 (13.5)  2801 (65.4) | 49 (15.8)  49 (15.8)  212 (68.4) | 901 **(21.1)**  585 (13.7)  2789 (65.2) | 53 (16.6)  43 (13.4)  224 (70) |
| Education  Primary/ below  Secondary/ above | 2368 (51.7)  2213 (48.3) | 2178 (51.0)  2094 (49.0) | 190 **(61.5)**  119 **(38.5)** | 2170 (50.9)  2092 (49.1) | 198 (62.1)  121 (37.9) |
| Grades A-B | 2172 (47.3) | 2087 (48.6) | 85 (27.6) | 2083 (48.7) | 89 (28.0) |
| Grade C | 783 (17.0) | 740 (13.7) | 43 (14.0) | 739 (17.3) | 44 (13.8) |
| Grade D/F | 1641 (35.7) | 1461 (34.1) | 180 **(58.4)** | 1456 (34.0) | 185 **(58.2)** |

Data are presented as mean ± SD for continuous variables and frequency (% of total N in column). COPD (chronic obstructive pulmonary disease)/asthma, cardiac disease and strokes were self-reported conditions to baseline questionnaire. Grades are quality grade for spirometry effort provided by the EasyOne spirometer.

**Table C.** Bland-Altman mean differences and limits of agreement (LoA) between spirometers by grades.

|  | **Grade A**  (FEV_1_=1,555, FVC=1,551) | **Grade B**  (FEV_1_=611, FVC=611) | **Grade C**  (FEV_1_=779, FVC=776) | **Grade D/F**  (FEV_1_=1637, FVC=1624) |
| --- | --- | --- | --- | --- |
| **FEV_1_, ml** | **-39** (-58.2, -19.1) | **-15** (-15.5, 44.8) | **-17** (-49, 15.8) | **-67** (-100, -34.7) |
| **LOA** | -811, 734 | -729, 759 | -924, 890 | -1391, 1256 |
| **FEV_1_, %** | **-1.2%** (-2.2, -0.2) | **1.2%** (-0.2, 2.7) | **-0.2%** (-1.8, 1.4) | **-3.4%** (-5.2, -1.7) |
| **LOA** | -39.4, 36.9 | -35.9, 38.4 | -45.9, 45.5 | -72.6, 65.7 |
| **FVC ml** | **-27** (-55.3, 1.9) | **45** (-1, 89.3) | **12** (-34.6, 59) | **95** (50.7, 140) |
| **LOA** | -1152, 1099 | -1044, 1134 | -1289, 1313 | -1702, 1893 |
| **FVC, %** | **-0.01%** (-1.0, 1.0) | **2.7%** (-1.1, 4.4) | **1.7%** (-0.02, 3.4) | **4.5%** (2.8, 6.2) |
| **LOA** | -42.3, 42.3 | -38, 43.6 | -45.8, 49.2 | -64.8, 73.9 |

- As suggested by the reviewer, we plotted the Bland Altman plot of the overall data with quality grades highlighted in different colors. As shown below, there were significant overlap of the data with grades A to C (color coded in black [grade A], blue [grade B], green [grade C]). Many of the data points, outside the LoA were mainly grades D/F (coded red). However, even among the acceptable grades (A-C), there were a few individuals with large differences between measurements falling outside the LoA.

The authors present data from 4,603 participants across 18 countries and 7 regions. The demographics of this population is presented in Table 1. Table 4 is based on data from >100,000 individuals which are not demographically reported, it is not clear whether there is overlap of individuals between MicroGP and EasyOne, and it would be interesting to perform sensitivity analysis in those individuals with data from both spirometers. It would also be possible to then specify spirometer in models as a covariate to see whether there is a significant association.

- The risk exposure-Lung function analysis has been removed from this revised paper. This was an interim analysis from the larger PURE study and therefore the sample sizes for data collected using the old and new spirometer were not balanced. Furthermore the methodology for this analysis is not well detailed in the current paper and to add this information to the current paper will substantially increase the length of the paper. Finally, we are planning to submit the risk exposure-lung function association as a full paper and therefore we want to avoid the risk of duplication. For these reasons, we have removed this data from the revised version of this paper. However, we will leave it up to the discretion of the editor, to let us know if by adding this data will strengthen the current work, which has already been expanded to include other analyses in the revised form. If this is the case, we will be happy to add this data back into the paper.

The authors could present supplemental information that the first five consecutive participants from each community are representative of the wider PURE study, which would also support Table 4 interpretation.

- The baseline characteristics of the larger PURE study of 100,000 individuals excluding those in the substudy are presented in Appendix II. Compared to the larger PURE study, the substudy (Table 1) showed a trend to higher rates of comorbidities including COPD/asthma and cardiac disease; and lower education level. Otherwise, for all other variables examined, the 2 populations were comparable in baseline characteristics.

Can the authors comment on the possibility of misclassification of COPD and asthma, 7% seems potentially low for COPD and asthma as a single variable. Were numbers representative across all regions, or do they reflect specific ones? In supplement, the authors could present a breakdown of strata by region to assist in interpretation of Table 3.

- The reported prevalence of COPD and asthma was based on self-reported response to the baseline questionnaire, which asked whether participants were aware of a diagnosis of COPD or asthma. We agree that this number is low but not surprising, since under-diagnosis of COPD/asthma has been reported to be high in many previous studies conducted mainly in HIC. This is likely to also be true in MIC and LIC, where access to healthcare is more limited and difficult. We have added a breakdown on the prevalence of self-reported known COPD/asthma by region in Table 1, which highlight the variation in prevalence across regions.

The authors should confirm whether assumptions for intraclass coefficients are achieved, such as those regarding normality and variance, as the ICC can be sensitive to this.

- Given the large sample size, any normality statistical testing will likely be significant. Therefore, we assessed the assumption of normality and constant variance by visually inspecting the histograms and plotting the residuals against the fitted values. This information has been added to the methods/statistical section.

The authors demonstrate good correlation and agreement overall, but can the authors comment on whether studies utilising a mixture of spirometers should apply weightings according to certain (demographic?) factors to support standardisation that may enable more accurate data alignment, in particular whether this valuable dataset can estimate such weightings, or whether the authors are confidence this would not be necessary. Supplementary sensitivity analysis of e.g. grade A-C repeat measures within a spirometer vs between spirometer may give further reliable insight in to comparability of datasets, coefficient of variation may facilitate this.

- Mean differences between spirometers across regions were generally small for FEV1 and showed no consistent bias. There were slightly larger mean differences between spirometers for FVC in some regions such as the Middle East and South America but again no consistent biases were observed across regions.
- We have provided more data according to grades (Table 4, Appendix III), which showed that mean differences between spirometers were small (within acceptable limits for between effort variability within individual) for both FEV_1_ and FVC even for the lower grades D/F. However, the limits of agreement showed an increase with lower quality grade efforts, suggesting greater variability between spirometers with lower grades.
- Even with somewhat wider limits of agreement, the 2 spirometers demonstrated sufficient precision in estimating the group means which were closely correlated and showed small mean differences. Since there was no systematic bias, it would be difficult to apply any ‘adjustments’ or ‘weighting’ to the spirometer particularly at the region, country or center levels, as these levels seemed to have contributed minimally to the variability between spirometers. As far as dealing with group means which is important for epidemiological studies, we feel there is no need to adjust for these small differences between spirometer, which appear to be random in nature. Furthermore, transforming the data using the GLI normative values, showed no meaningful difference between the group mean Z-scores.

There are a few minor typographical errors, for example Table 3 includes 95%CI with some missing or erroneous symbols. E.g. FVC No COPD/Asthma mean Diff % -2.5 (1.6, 3.3): is this a negative difference with erroneous 95%CI or positive difference? Similarly for FEV1 age >65 and 50-65 mean diff %. There may be some issues with 95%CI values for females FVC and males FEV1 that authors should check. Table 4 adjusted model EasyOne-FVC -22,8 rather than -22.8

- These have been addressed.

*************************************************************************************************

**Reviewer #3:** Summary
The authors compared the correlation and agreement between two spirometry devices (MicroGP and EasyOne) in a subset of 4603 participants from the PURE cohort study. Device allocation was random and both tests performed on the same day. Appropriate statistical methods were chosen including Intra-class correlation coefficients and Bland-Altman plots. Results were stratified by clinical and epidemiological characteristics and presented appropriately in figures and tables. Strong correlations were noted between paired FEV1 and FVC measurements, and differences between spirometers were largely within the limits of between effort reproducibility according to ATS/ERS guidelines. Limits of agreement were wide, especially for FVC. Participant characteristics appeared to have minimal impact on agreement between the two devices, however more variation was noted by location especially for South America and the Middle East, which may be clinically significant for FVC. Robust discussion of strengths and limitations was presented. The authors concluded that the use of these two spirometers provide reliable data that are without bias. This conclusion while understandable as the results from the spirometers are highly correlated, is perhaps a little overstretched on account of the wide limits of agreement.

Major concerns

The authors do not provide enough detail on exactly how the random order was generated when deciding which spirometer to use first? This is import to avoid a training effect.

- The order of spirometer measurements was randomly generated by the coordinating site (in Hamilton, Canada) and issued to the centers prior to the data collection. This information has been added to the methods section.

The limits of agreement for FEV1 and FVC are wide and the authors provide justification for this based on previous research in studies with large sample sizes. However, it may be an over stretch to conclude that “Our findings suggest that the use of these two spirometers in large long-term studies provide reliable data that are without bias.” The fact the limits of agreement are wide, suggests some ambiguity in the result especially for FVC, which is likely clinically significant in some of the populations and caution should be taken when forming conclusions.

- Spirometry testing is inherently a variable measurement which requires maximal effort of a relatively complex maneuver from the participant/ patients. This is unlike any other physiological measurements which are taken passively while patients are at rest. There are patient, technician/ instructor and device factors, which contribute to the variability between measurements. Consequently, the standard or reference method has always been for spirometry to be measured in highly controlled settings such as dedicated pulmonary function laboratories in tertiary centers, which substantially limit the access of this important measurement to the broader population. Even under controlled conditions, up to 1/3 of measurements are deemed unacceptable or non-reproducible according to ATS/ERS standards. There are 2 factors which determine whether participants are likely to achieve acceptable and reproducible measurements. These are the ability to achieve maximal effort and to maintain maximal effort to the end of expiration. It is much easier to achieve maximal effort for a short period of time e.g. such as the effort needed to generate FEV1, as appose to maintaining maximal effort to the end of expiration that is needed for FVC, (which may take up to 7 seconds or more in adults). Therefore, it is not surprising to see that the precision and agreement in the FEV1 measurement was better than for FVC. Given the inherent variability of these measurements, a 95% LoA is expected to be wide. While it is always better to have smaller LoA, there is presently, no recommendations for the range of LoA. Furthermore, given the diverse population and large differences in anthropometrics; we recommend placing more emphasis on the relative differences or LoA in relative terms as this takes into consideration differences between populations in lung size/volume. In our opinion, the ability of these instruments to provide sufficient precision in the estimates of group means that were highly correlated with small and non-meaningful differences across diverse settings in a real-world setting, is an important finding and support valid comparisons between group means between spirometers at a population level.

Minor issues

It would be appropriate in this circumstance to provide a brief description in the methods section as to how the two spirometers work and how they are different. For example, ultrasonic spirometers measure flow independently of gas composition, pressure, temperature, and humidity, and why the two spirometers may be expected to produce different results. This would help give context as to why it is important to compare these two devices.

- This information on spirometers has been added to the methods section.

The authors stated that 65% of participants achieved clinically acceptable measurements using the automated grading criteria generated by the EasyOne device, do they have data from the MicroGP from which you can classify spirometry manoeuvres into these ranges? It is important to know how many participants achieved clinically acceptable results on each device if they are going to quote the 65% value from the EasyOne in the publication. This speaks to the ease of use of the monitors.

- The old microGP spirometer did not have the capacity to generate quality grades. However, lung function measurements from the 2 spirometers were collected within a short time interval and in a random order, which could facilitate similar and consistent efforts across the spirometers.

It may be useful to include mean percent predicted values for each device (e.g. FEV1, EasyOne 98% of predicted, FEV1 MicroGP 96% of predicted). This will help to clearly display how the difference between devices impacts on the predicted norm for each population.

- We have provided the Z scores using GLI normative values to Table 1 in the revised manuscript, rather than the %pred value since a difference of <0.5 SD is generally considered to be non-significant.
